# *Staphylococcus aureus *intestinal colonization is associated with increased frequency of *S. aureus *on skin of hospitalized patients

**DOI:** 10.1186/1471-2334-7-105

**Published:** 2007-09-11

**Authors:** Anita Bhalla, David C Aron, Curtis J Donskey

**Affiliations:** 1 Research Service, Louis Stokes Cleveland Veterans Affairs Medical Center, 10701 East Blvd., Cleveland, Ohio, USA; 2 Center for Quality Improvement Research, Louis Stokes Cleveland Department of Veterans Affairs Medical Center, Cleveland, Ohio, USA

## Abstract

**Background:**

Intestinal colonization by *Staphylococcus aureus *among hospitalized patients has been associated with increased risk of staphylococcal infection and could potentially contribute to transmission. We hypothesized that *S. aureus *intestinal colonization is associated with increased frequency of *S. aureus *on patients' skin and nearby environmental surfaces.

**Methods:**

Selected inpatients were cultured weekly for *S. aureus *from stool, nares, skin (groin and axilla), and environmental surfaces (bed rail and bedside table). Investigator's hands were cultured after contacting the patients' skin and the environmental surfaces.

**Results:**

Of 71 subjects, 32 (45.1%) had negative nares and stool cultures, 23 (32.4%) had positive nares and stool cultures, 13 (18.3%) were nares carriers only, and 3 (4.2%) were stool carriers only. Of the 39 patients with *S. aureus *carriage, 30 (76.9%) had methicillin-resistant isolates. In comparison to nares colonization only, nares and intestinal colonization was associated with increased frequency of positive skin cultures (41% versus 77%; p = 0.001) and trends toward increased environmental contamination (45% versus 62%; p = 0.188) and acquisition on investigator's hands (36% versus 60%; p = 0.057). Patients with negative nares and stool cultures had low frequency of *S. aureus *on skin and the environment (4.8% and 11.3%, respectively).

**Conclusion:**

We found that hospitalized patients with *S. aureus *nares and/or stool carriage frequently had *S. aureus *on their skin and on nearby environmental surfaces. *S. aureus *intestinal colonization was associated with increased frequency of positive skin cultures, which could potentially facilitate staphylococcal infections and nosocomial transmission.

## Background

*Staphylococcus aureus *is an important cause of community-acquired and healthcare-associated infections [1]. The nose (anterior nares) is considered the primary site of colonization with *S. aureus*; however, several recent studies suggest that colonization of the intestinal tracts of hospitalized patients may have important clinical implications [2-4]. We found that more than half of patients with vancomycin-resistant *Enterococcus *(VRE) stool colonization had coexisting intestinal colonization with *S. aureus*, providing a potential reservoir for the emergence vancomycin-resistant *S. aureus *isolates [2]. Boyce et al. [3] and Gravet et al. [4] have suggested that enterotoxin-producing methicillin-resistant *S. aureus *(MRSA) strains may be an underappreciated cause of antibiotic-associated diarrhea. Finally, Squier et al. [5] found that intensive care and liver transplant unit patients with both rectal and nares MRSA colonization had significantly higher rates of *S. aureus *infection than did patients with nares carriage alone (40% vs. 18%).

The mechanism by which intestinal colonization by *S. aureus *might lead to an increased risk of staphylococcal infections is not known. Squier et al. [5] proposed that intestinal colonization by *S. aureus *could be associated with increased frequency of colonization or contamination of skin sites, thereby increasing the risk for contamination of devices, wounds, and mucous membranes. In addition to facilitating infections, shedding of large number of *S. aureus *from stool onto skin and environmental surfaces could potentially contribute to nosocomial transmission [5-7]. Other explanations for the association between intestinal colonization with *S. aureus *and infections are also possible. For example, strains with increased virulence might have a greater propensity to colonize the intestinal tract or the skin. We performed a prospective observational study to test the hypothesis that intestinal colonization by *S. aureus *is associated with increased frequency of positive skin cultures and increased contamination of nearby environmental surfaces.

## Methods

### Setting and study design

We performed a 6-month prospective study of selected inpatients at the Cleveland Veterans Affairs Medical Center. Subjects were selected by reviewing in sequence a listing of all inpatients hospitalized on the first workday of each week; the list was generated based on time of admission. Patients with an anticipated duration of additional stay in the hospital of less than 3 days were excluded in order to allow for collection of serial samples from a significant proportion of the subjects. After oral informed consent was obtained, stool samples were collected each week during the admission. Stool samples were refrigerated at 4°C and either processed within one week, or frozen at (-) 80°C for analysis at a later date. We tested for the presence of *S. aureus *in stool specimens, and if present, determined the density of colonization. Patients were considered to have persistent stool or nares colonization if *S. aureus *was cultured from three or more consecutive stool or nares samples, respectively.

Within 1 day of each stool collection, cultures were obtained from the patient's anterior nares, skin (groin and axilla), and environment (bed rail and bedside table) using pre-moistened cotton-tipped swabs. In addition, hand imprint cultures for *S. aureus *were obtained after contacting the same skin and environment sites noted above as described previously [8]. In short, the investigators disinfected their hands with 62.5% alcohol hand rub and imprinted 1 hand onto a mannitol salt agar (Becton Dickinson, Cockeysville, MD) plate to confirm that no *S. aureus *were present. The same hand was then placed sequentially onto the patient's bedrail, bedside table, groin, and axilla, each for 5 seconds. The fingertips and palms were then imprinted onto a second mannitol salt agar plate and processed as described below.

Information regarding demographic characteristics, coexisting illnesses, *S. aureus *infections, and medications and treatments was obtained through standardized medical record review. All subjects were assessed for development of *S. aureus *infections during their admissions and charts were reviewed to evaluate whether infections occurred within 90 days after discharge. Because some antibiotics have in vitro inhibitory activity against *S. aureus *strains, we assessed whether therapy with antibiotics with in vitro activity against the colonizing strains was associated with elimination of nares or intestinal carriage. Infections were defined using the Centers for Disease Control and Prevention criteria for nosocomial infections [9]. The Louis Stokes Cleveland Department of Veterans Affairs Medical Center's Institutional Review Board approved the study protocol.

### Microbiologic analysis and molecular typing

In order to screen for the presence of *S. aureus*, samples were plated onto mannitol salt agar (Becton Dickinson). Plates were incubated at 37°C for 48 hours and colonies consistent with *S. aureus *were subjected to identification and susceptibility testing in accordance with National Committee for Clinical Laboratory Standards guidelines [10]. The density of organisms/g of stool was determined as previously described [2]. If no organisms were detected, the lower limit of detection was assigned (~1.5 log/g). The number of colonies of *S. aureus *from cultures of nares, skin, environmental surfaces, and investigator's hands were counted. Pulsed-field gel electrophoresis was performed on selected *S. aureus *isolates using a modification of the technique of Hoyen et al [11]. The plugs were digested with *Sma*I for 16 hours (Promega, Madison, Wis.). Pulsed-field gel patterns were interpreted using the criteria of Tenover et al [12].

### Statistical analysis

Data were analyzed using SPSS version 10.0 (Chicago, IL). We compared the characteristics of 3 groups (i.e., stool colonization with or without concurrent nares colonization, nares colonization only, and no colonization as evidenced by negative nares and stool cultures). Patients with positive stool cultures but negative nares cultures were not analyzed separately for differences in patient characteristics because there were only 3 subjects in this group. One-way analysis of variance was used for analysis of continuous variables and the Pearson Chi-square test or Fisher's exact test was used for analysis of categorical data. Additional bi-variate analyses were performed to compare characteristics of patients with any *S. aureus *colonization of nares or stool to those with no colonization (i.e., negative nares and stool cultures), and to compare colonized patients with stool carriage (with or without concurrent nares carriage) to patients with nares colonization only. For purposes of analysis, patients with a positive nares or stool culture at any time during the study were considered to be positive at these sites. The frequencies of positive cultures for skin, environment, and hand acquisition cultures were analyzed using the Pearson Chi-square test or Fisher's exact test. All reported p values are two-sided. Unless otherwise stated, mean values are given as means ± SD. Finally, the impact of antibiotic treatment was examined to determine whether antibiotics with in vitro activity against colonizing *S. aureus *strains would inhibit nares or intestinal colonization.

## Results

### Characteristics of the patients

Seventy-one total patients were enrolled in the study. Thirty-two (45.1%) patients had negative nares and stool cultures for *S. aureus*; 23 (32.4%) had positive nares and stool cultures; 13 (18.3%) had positive nares but negative stool cultures; and 3 (4.2%) had negative nares but positive stool cultures. Of the 39 patients with *S. aureus *colonization, 30 (76.9%) had MRSA. Of the 23 patients with nares and intestinal colonization, 18 (78.3%) had 3 sets of cultures obtained and 100% had persistent positive stool and nares cultures (i.e. positive cultures from 3 consecutive cultures). The mean density of *S. aureus *in stool of these patients was 4.39 log/g of stool (range, 1.5 to 7.4 log/g). Fifteen of the 18 patients with persistent positive stool cultures maintained *S. aureus *colonization for the duration of their admission and/or participation in the study (range, 3 to 22 weeks). Five of the 13 patients with positive nares but negative stool cultures had 3 consecutive sets of cultures, and 4 of 5 (80%) had persistent positive nares cultures.

When patients with stool carriage (with or without concurrent nares carriage), nares carriage only, and no stool or nares carriage were compared (Table 1), only the development of *S. aureus *infections was significantly different among the 3 groups. When patients with any *S. aureus *colonization were compared to those with no stool or nares carriage, those with *S. aureus *colonization were more likely to have diarrhea or fecal incontinence (59.0% versus 25.0%; p = 0.02), *S. aureus *infection (25.6% versus 3.1%; *P *= 0.009), and increased length of stay (21.5 ± 17.7 versus 13.2 ± 12.1; p = 0.02); there was a trend toward increased frequency of wounds in patients with any *S. aureus *colonization versus those with no carriage (38.5% versus 18.8%; p = 0.07). Patients with stool carriage (with or without concurrent nares carriage) did not differ significantly from patients with nares carriage only in any of the variables assessed.

**Table 1:** Characteristics of the 71 study patients and events during the study *Staphylococcus aureus *colonization sites

	***Stool +*** ***Nares +/-***	***Nares*** ***only***	***Nares -*** ***Stool -***	
**Characteristic**	**(N = 26)**	**(N = 13)**	**(N = 32)**	p*
**At baseline**				
Age, years, mean ± SD	65.0 ± 14.5	66.1 ± 11.4	63.1 ± 13.2	0.76
Length of stay, mean ± SD	22.6 ± 18.6	19.2 ± 16.1	13.2 ± 12.1	0.13
Male sex, no. (%)	26 (100)	13 (100)	32 (100)	1
Clinical conditions, no. (%)				
Chronic renal failure	3 (11.5)	2 (15.4)	2 (6.3)	0.61
Diabetes mellitus	9 (34.6)	4 (30.8)	12 (37.5)	0.91
Chronic dermatologic conditions	1 (3.8)	1 (7.7)	1 (3.1)	0.25
Wounds	11 (42.3)	4 (30.8)	6 (18.8)	0.15
Liver disease	4 (15.4)	1 (7.7)	1 (3.1)	0.25
Cancer	5 (19.2)	0 (0)	7 (21.9)	0.19
Nursing home resident, no. (%)	4 (15.4)	3 (23.1)	5 (15.6)	0.81
**During the study, no. (%)**				
Admission to intensive care unit	4 (15.4	1 (7.7)	2 (6.3)	0.13
Nasogastric tube	6 (23.1)	2 (15.4)	3 (9.4)	0.36
Mechanical ventilation	6 (23.1)	1 (7.7)	2 (6.3)	0.13
Antibiotic therapy	18 (69.2)	9 (69.2)	21 (65.6)	0.95
Vancomycin-resistant *Enterococcus *colonization	10 (38.5)	4 (30.8)	7 (21.9)	0.39
Central venous catheter	5 (19.2)	1 (7.7)	6 (18.8)	0.62
Diarrhea or fecal incontinence	15 (57.7)	8 (61.5)	8 (25)	0.06
Surgery	4 (15.4)	2 (15.4)	6 (18.8)	0.93
Proton pump inhibitor or H_2 _blocker	18 (69.2)	9 (69.2)	21 (65.6)	0.95
*S. aureus *infection	8 (30.8)	2 (15.4)	1 (3.1)	0.02

*p values refer to overall differences among the 3 groups

Overall, 15.5% of the study patients (11 of 71) developed *S. aureus *infections. These included bacteremia in 5 patients, empyema in 1, pneumonia in 3, and wound infection in 2. *S. aureus *infection occurred in 30.4% of patients with nares and stool colonization (7 of 23), 15.4% of patients with nares colonization only (2 of 13), 33.3% of patients with stool colonization only (1 of 3), and in 3.1% of those with no stool or nares colonization (1 of 32). *S. aureus *infection developed more often in patients with stool colonization (8/26; 30.8%) versus those with only nares colonization (2/13; 15.4%) but the difference was not statistically significant (p = 0.30).

### Contamination of skin, environment, and hands with S. aureus

**Figure 1:**
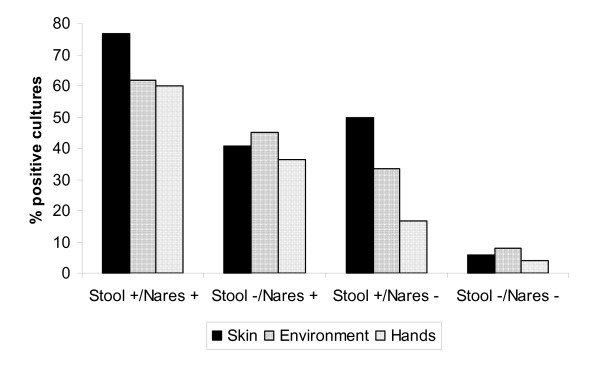
Percentage of positive cultures of skin, environment (bed rails and bedside table) and investigator's hands for *Staphylococcus aureus *among hospitalized patients with nares and stool colonization, nares colonization only, stool colonization only, or no *S. aureus *carriage. For purposes of analysis, patients with a positive nares or stool culture at any time during the study were considered to be positive at these sites. Investigator's hand cultures were obtained by placing a hand sequentially on the patient's skin, bed rail, and bedside table followed by imprinting onto a mannitol agar plate.

A total of 138 sets of cultures were obtained (mean, 1.9 per patient; range, 1 to 6). Figure 1 provides a summary of the results. Patients with *S. aureus *nares and intestinal colonization or nares colonization only were more likely to have positive cultures of skin and environment than patients who had negative nares and stool cultures (p < 0.001), and investigators were more likely to acquire *S. aureus *on hands after contacting skin and environmental surfaces of these patients (p < 0.001). Patients with nares and intestinal colonization were more likely than those with nares colonization only to have positive skin cultures (p = 0.001). In comparison to patients with nares colonization only, patients with nares and intestinal colonization also had non-significant trends toward increased *S. aureus *contamination of environmental surfaces (p = 0.188) and increased acquisition of *S. aureus *on investigator's hands (p = 0.057). Patients with negative nares but positive stool cultures had trends toward higher rates of skin, environment, and investigator hand cultures than patients with negative cultures, but these differences were not statistically significant (p ≥ 0.05). However, only 3 patients and 6 sets of cultures were included in this group, providing relatively little power to determine if significant differences were present. Among the patients with intestinal colonization, the 15 subjects with fecal incontinence or diarrhea had non-significant trends toward higher rates of skin (75% versus 65.9%, respectively) and environmental (59.1% versus 50%, respectively) contamination than the 11 subjects with no fecal incontinence or diarrhea (p ≥ 0.05).

Of the 138 sets of cultures, 61 (44.2%) sets obtained from 28 patients (39.4% of all patients) had positive skin cultures. The axilla and groin sites were positive in 26 of the 61 (43%) sets of positive cultures, only the groin was positive in 32 of 61 (51.6%) sets, and only the axilla was positive in 3 (4.9%). The number of colonies of *S. aureus *obtained from the groin and axilla was frequently too numerous to count. Positive environmental cultures yielded a mean of 12.7 colonies of *S. aureus *(range, 1 to 80). Positive investigator hand cultures after contact with skin and environmental surfaces yielded a mean of 15.3 colonies of *S. aureus *(range, 1 to 80).

### Effect of antibiotics with in vitro inhibitory activity against colonizing strains

**Figure 2:**
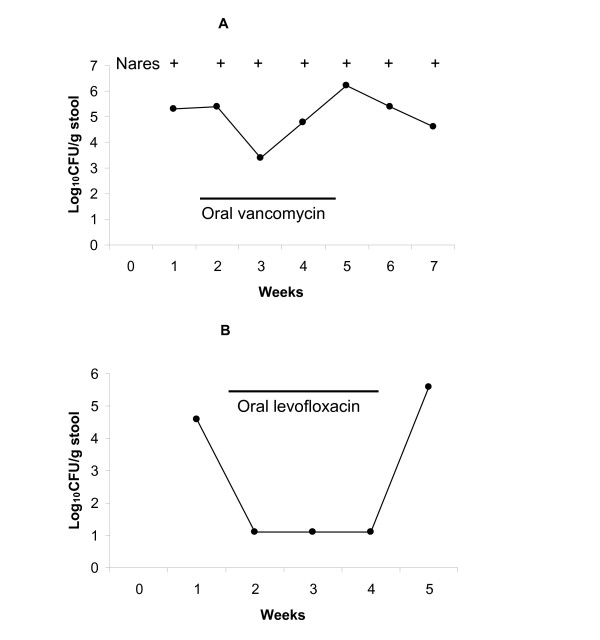
Effect of antibiotic therapy with agents with in vitro inhibitory activity against colonizing methicillin-resistant *Staphylococcus aureus *(MRSA) strains. One patient (A) received oral vancomycin for treatment of *Clostridium difficile*-associated diarrhea and a second (B) received oral levofloxacin for treatment of a urinary tract infection. The minimum inhibitory concentration (MIC) of vancomycin for the MRSA isolate in A was 0.25 μg/mL; the MIC of levofloxacin for the MRSA isolate in B was 0.125 μg/mL). + = positive nares culture; - = negative nares culture; solid circles = density of MRSA in stool.

Four patients with nares and intestinal *S. aureus *susceptible to vancomycin continued to have colonization of both sites while receiving therapy with intravenous vancomycin; a fifth patient from this group maintained stool colonization with MRSA while receiving oral vancomycin therapy for *C. difficile *infection (Figure 2A). One patient with nares MRSA colonization only continued to have positive nares cultures while receiving intravenous vancomycin. One patient with nares and intestinal colonization with a levofloxacin-susceptible MRSA isolate developed negative cultures at both sites while receiving oral levofloxacin (Figure 2B). However, 5 patients colonized with levofloxacin-resistant *S. aureus *isolates maintained persistent nares and/or stool cultures during therapy with this agent. No patients received therapy with other agents with in vitro inhibitory activity against colonizing *S. aureus *strains.

### Molecular typing

For 8 study patients, pulsed-field gel electrophoresis was performed on multiple *S. aureus *isolates. For 4 patients with nares, stool, skin, and environmental isolates, the nares, stool, and skin isolates of each patient were clonally identical; environmental isolates from 3 of the 4 patients were identical to isolates cultured from the other sites, whereas an environmental isolate from the fourth patient was unrelated to the isolates from the other sites. For 4 patients with nares, skin, and environmental isolates, the skin isolates were identical (3 of 4 patients) or closely related (1 of 4 patients) to the corresponding nares isolates; environmental isolates from 2 of the 4 patients were identical to isolates cultured from nares, whereas environmental isolates of the other 2 patients were unrelated to nares or skin isolates.

## Discussion

In this prospective study, we found that two-thirds of hospitalized patients harboring *S. aureus *had intestinal colonization with these organisms. Of the 26 study patients with *S. aureus *in stool, 20 (77%) had persistent stool carriage and 23 (88%) had concurrent nares carriage. These data are consistent with previous studies that have demonstrated that *S. aureus *intestinal colonization is common among hospitalized patients [2-5]. The major new finding of our study was that patients with nares and intestinal *S. aureus *colonization were significantly more likely than those with nares colonization only to have positive skin cultures, and these subjects exhibited a non-significant trend toward increased contamination of environmental surfaces and of acquisition on investigator's hands after contacting skin and environmental surfaces. Because staphylococci on skin may contaminate devices or wounds and be acquired on hands, our data provide support for the hypothesis that colonization of the intestinal tract may facilitate *S. aureus *infections and nosocomial transmission.

As noted previously, Squier et al. [5] found that intensive care and liver transplant unit patients with both rectal and nares MRSA colonization had significantly higher rates of *S. aureus *infection than did patients with nares carriage alone (40% vs. 18%). We also found that patients with nares and intestinal *S. aureus *colonization developed infections about twice as often as those with nares colonization alone (30.8% versus 15.4%), but this difference was not statistically significant. It should be noted, however, that the small numbers of subjects included in our study provided limited power to distinguish between the rates of infection in the 2 groups. Additional studies are indicated to examine the association between *S. aureus *intestinal colonization and the development of infections.

Previous studies suggest that factors such as decreased gastric acidity, nasogastric tubes, and antibiotic therapy may contribute to the development of intestinal colonization by *S. aureus *[2,3,13]. We did not find a significant association between these factors and *S. aureus *intestinal colonization in our study. It is notable that more than half of the patients with nares *S. aureus *colonization only received treatment with proton pump inhibitors and antibiotics, suggesting that these factors alone may not be sufficient for the development of intestinal colonization in many patients with nares carriage. Because antibiotics may also inhibit colonization by pathogens, we examined the impact of treatment with agents with in vitro inhibitory activity against *S. aureus*. Intravenous vancomycin had no apparent effect on nares or intestinal *S. aureus *colonization, possibly due to the fact that relatively low concentrations of this agent are secreted into nares passages or into the intestinal tract during parenteral administration [14]. In one patient, oral levofloxacin temporarily was associated with loss of detection of nares and intestinal colonization with a levofloxacin-susceptible MRSA strain; levofloxacin therapy did not result in loss of colonization by levofloxacin-resistant *S. aureus *strains.

Two recent European studies have evaluated the use of oral vancomycin therapy as a means to eliminate MRSA intestinal colonization [15,16]. In one study, oral vancomycin therapy was associated with a significant reduction in methicillin-resistant *S. aureus *infections [15]. Interestingly, one patient in our study maintained intestinal colonization with MRSA during therapy with oral vancomycin (Figure 2A), which results in high concentrations in the colon. In mice, we found that intestinal colonization with 1 of 2 MRSA strains also was not inhibited by oral vancomycin; we hypothesized that such persistence might be due to growth of MRSA within a biofilm in the colonic mucus layer [13]. Further studies are needed to clarify the potential for eradication of intestinal *S. aureus *colonization with oral non-absorbed antibiotics.

Our study has several limitations. First, our study population may not be representative of all patient populations because only men were included and we excluded patients with an anticipated additional length of hospital stay of less than 3 days. Second, 15 of 26 (58%) patients with intestinal *S. aureus *colonization had diarrhea or fecal incontinence during the period of the study which is likely to have contributed to shedding of organisms onto skin and into the environment. It is possible that continent patients with no diarrhea may be less likely to shed *S. aureus*, and we observed a trend toward lower rates of skin and environmental *S. aureus *among these patients in comparison to patients with fecal incontinence or diarrhea. Third, hand cultures were obtained after contact with both skin and environmental sites, and therefore it is not possible to determine the relative contribution of skin or the environment to hand acquisition. Fourth, *S. aureus *isolates cultured from surfaces may have been shed by previous patients occupying the study patient's rooms because staphylococci may persist for long periods on surfaces. The fact that some environmental isolates were clonally unrelated to the study patient's nares and stool isolates suggests that contamination may have come from previous room occupants or from hands of transiently colonized healthcare workers, or that patient and environmental cultures may be polyclonal in nature. Fifth, Boyce et al. [17] found that the presence of MRSA in wounds or urine was associated with increased environmental contamination. In our study, there was a non-significant trend toward increased frequency of wounds in patients with stool and/or nares colonization than those with nares colonization only (42.3% versus 30.8%); however, the frequency of wounds from which *S. aureus *was isolated from clinical cultures was similar in both groups (7 of 26 {26.9%} and 3 of 13 {23.1%}), respectively. Finally, although we propose that the increased isolation of *S. aureus *from skin of patients with intestinal colonization was attributable to fecal contamination, it is possible that strains with an increased propensity to colonize the intestinal tract also have an increased tendency to colonize or contaminate skin.

## Conclusion

We found that hospitalized patients with *S. aureus *carriage had high rates of positive skin cultures and of contamination of environmental surfaces. Patients with nares and intestinal *S. aureus *colonization were significantly more likely than those with nares colonization only to have positive skin cultures, and these subjects exhibited a non-significant trend toward increased contamination of environmental surfaces and of acquisition on investigator's hands after contacting skin and environmental surfaces. Further research is needed to better define the significance of intestinal colonization with staphylococci with regard to the pathogenesis of *S. aureus *infections and nosocomial transmission.

## Competing interests

The author(s) declare that they have no competing interests.

## Authors' contributions

CJD conceived of the study, participated in drafting the manuscript, and edited the manuscript. AB performed the cultures and data collection and assisted in drafting the manuscript. DCA performed the statistical analyses and assisted in editing the manuscript.

## Pre-publication history

The pre-publication history for this paper can be accessed here:


